# Bis(4-amino-3,5-di-2-pyridyl-4*H*-1,2,4-triazole)diaquanickel(II) bis(perchlorate)

**DOI:** 10.1107/S1600536809012598

**Published:** 2009-04-10

**Authors:** Chun-Fu Shao, Li-Chuan Geng

**Affiliations:** a College of Chemistry and Life Science, Tianjin Normal University, Tianjin 300387, People’s Republic of China

## Abstract

In the mol­ecular structure of the centrosymmetric mononuclear complex [Ni(2-bpt)_2_(H_2_O)_2_](ClO_4_)_2_ [2-bpt = 4-amino-3,5-di-2-pyridyl-1,2,4-triazole, (C_12_H_10_N_6_)], the central Ni^II^ atom is six-coordinated by a pair of chelating 2-bpt ligands and two water mol­ecules. Inter­molecular O—H⋯N inter­actions link the monomeric units into a two-dimensional hydrogen-bonded (4,4) network, which is extended to a three-dimensional supra­molecular aggregate *via* π⋯π stacking inter­actions [centroid–centroid distances 3.809 (3) and 3.499 (3)  Å].

## Related literature

Diverse coordination architectures can be constructed by coordinative bonds using metal ions to combine with multifunctional ligands, see: Moulton & Zaworotko (2001 ▶). Supra­molecular inter­actions such as hydrogen bonding and aromatic stacking are usually used to extend or sustain the resultant structures, see: Roesky & Andruh (2003 ▶); Ye *et al.* (2005 ▶); Du *et al.* (2007 ▶). For polypyrid­yl–transition metal complexes, see: Haasnoot (2000 ▶). For the potential ability of 4-amino-3,5-di-2-pyridyl-1,2,4-triazole (2-bpt) to provide multi-coordination modes and generate hydrogen-bonding and/or aromatic stacking inter­actions, see: Van Koningsbruggen *et al.* (1998 ▶); Moliner *et al.* (2001 ▶); García-Couceiro *et al.* (2004 ▶); Peng *et al.* (2006 ▶). For Ni^II^–2-bpt complexes, see: Keij *et al.* (1984 ▶); Tong *et al.* (2007 ▶). For the (4,4) topology, see: Batten & Robson (1998 ▶).
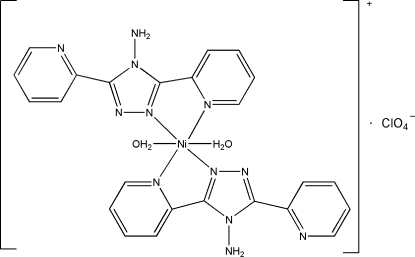


## Experimental

### 

#### Crystal data

[Ni(C_12_H_10_N_6_)_2_(H_2_O)_2_](ClO_4_)_2_
*M* *_r_* = 770.16
Monoclinic, 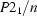

*a* = 9.9219 (15) Å
*b* = 14.359 (2) Å
*c* = 10.9220 (18) Å
β = 100.560 (3)°
*V* = 1529.7 (4) Å^3^
*Z* = 2
Mo *K*α radiation
μ = 0.89 mm^−1^
*T* = 296 K
0.20 × 0.18 × 0.16 mm

#### Data collection

Bruker SMART CCD area-detector diffractometer
Absorption correction: multi-scan (*SADABS*; Bruker, 2001 ▶) *T*_min_ = 0.840, *T*_max_ = 0.870
7639 measured reflections
2686 independent reflections
2171 reflections with *I* > 2σ(*I*)
*R*_int_ = 0.028

#### Refinement

*R*[*F* ^2^ > 2σ(*F* ^2^)] = 0.051
*wR*(*F* ^2^) = 0.154
*S* = 1.07
2686 reflections
223 parameters
H-atom parameters constrained
Δρ_max_ = 1.12 e Å^−3^
Δρ_min_ = −0.40 e Å^−3^

### 

Data collection: *APEX2* (Bruker, 2003 ▶); cell refinement: *SAINT* (Bruker, 2001 ▶); data reduction: *SAINT*; program(s) used to solve structure: *SHELXS97* (Sheldrick, 2008 ▶); program(s) used to refine structure: *SHELXL97* (Sheldrick, 2008 ▶); molecular graphics: *DIAMOND* (Brandenburg & Berndt, 1999 ▶); software used to prepare material for publication: *SHELXTL* (Sheldrick, 2008 ▶) and *PLATON* (Spek, 2009 ▶).

## Supplementary Material

Crystal structure: contains datablocks I, global. DOI: 10.1107/S1600536809012598/hg2489sup1.cif

Structure factors: contains datablocks I. DOI: 10.1107/S1600536809012598/hg2489Isup2.hkl

Additional supplementary materials:  crystallographic information; 3D view; checkCIF report

## Figures and Tables

**Table 1:** Hydrogen-bond geometry (Å, °)

*D*—H⋯*A*	*D*—H	H⋯*A*	*D*⋯*A*	*D*—H⋯*A*
O1—H1*A*⋯O3^i^	0.85	2.51	3.245 (8)	146
O1—H1*A*⋯O4^i^	0.85	2.11	2.918 (7)	158
O1—H1*B*⋯O2	0.85	2.44	3.085 (5)	134
O1—H1*B*⋯N6^i^	0.85	2.45	3.100 (5)	134
N5—H5*A*⋯O5^ii^	0.90	2.28	3.078 (7)	148
N5—H5*B*⋯N6	0.90	2.17	2.886 (5)	136

Symmetry codes: (i) 
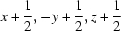
; (ii) 
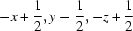
.

**Fig. 1.**
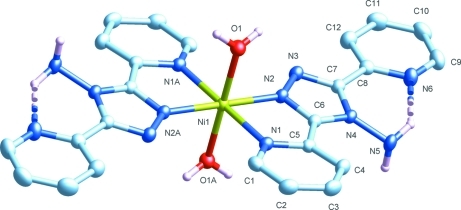
Representation of (I) with atomic labels of asymmetric unit and coordination sphere, shown with 30% probability displacement ellipsoids. Except for water and amino, all the hydrogen atoms as well as lattice ClO4- anion are omitted for clarity and the intramolecular hydrogen bonds are indicated by dashed lines. [Symmetry code A: - x + 1, - y, - z + 1]

**Fig. 2.**
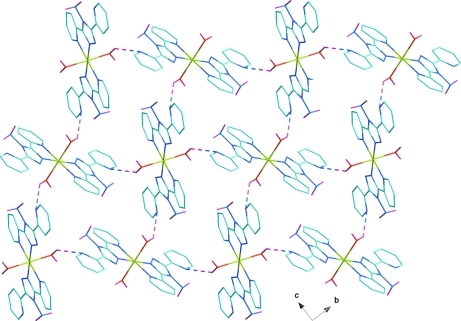
A perspective view of the two-dimensional hydrogen-bonded (4,4) net along the [011] plane.

**Fig. 3.**
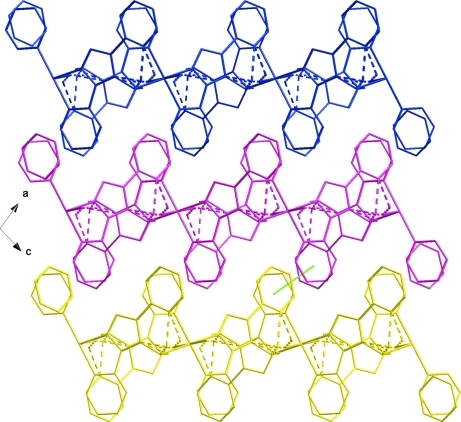
View of the 3-D supramolecular aggregate constructed from interlayer aromatic stacking interactions (each color represents a 2-D net and the green line indicates the aromatic stacking interaction).

## supplementary crystallographic information

### Comment

It is widely known that diverse coordination architectures can be constructed by coordinative bonds using metal ions to combine with multifunctional ligands (Moulton & Zaworotko, 2001). Aside from that, supramolecular interactions such as hydrogen bonding and aromatic stacking are usually used as the assistant tools to extend or sustain the resultant structures (Roesky & Andruh, 2003; Ye *et al.*,  2005; Du *et al.*, 2007). It has been reported that 1,2,4-triazole a is stronger σ-donor and weaker π-acceptor than 2,2'-bipyridine and their derivatives have gained considerable interest in recent years in the development of polypyridyl transition metal complexes (Haasnoot, 2000). Recently, one triazole derivative, 4-amino-3,5-di-2-pyridyl-1,2,4-triazole (2-bpt) has attracted our interest because of its potential ability for providing multi-coordination modes and generating hydrogen-bonding and/or aromatic stacking interactions (Van Koningsbruggen *et al.*, 1998; Moliner *et al.*, 2001; García-Couceiro *et al.*,2004; Peng *et al.*, 2006). With respect to the Ni^II^-2-bpt complexes, mononuclear and binuclear molecules have been reported (Keij *et al.*, 1984; Tong *et al.*, 2007), in which anions such as Cl^-^ and N_3_^-^ are existent, coordinating to the metal ion or serving as lattice entity to charge compensate. Here we report a new mononuclear Ni^II^-2-bpt complex [Ni(2-bpt)_2_(H_2_O)_2_](ClO_4_)_2_ (I), in which 2-bpt acts as a chelating reagent and supramolecular interactions such as hydrogen bonds and aromatic stacking can extend the mononuclear molecule into a three-dimensional architecture.

The molecular structure of (I) reveals a neutral centrosymmetric mononuclear complex, with the asymmetric unit of which comprising a half-occupied Ni^II^ atom, one 2-bpt molecule, one water ligand as well as one lattice ClO_4_^-^ anion. As depicted in Fig. 1, the distorted octahedral Ni^II^ center, which is located on a crystallographic inversion center, is defined by two pairs of chelating nitrogen donors from two individual 2-bpt molecules as well as two water ligands.  The axial Ni—N distances [2.037 (3) Å] are significantly shorter than those of the Ni—O and Ni—N equatorial lengths [2.101 (3) and 2.111 (3) Å]. In the structure, 2-bpt molecule exhibits *trans*-conformation and intramolecular N5—H5B···N6 hydrogen bond (Table 2) can be detected between the adjacent amino and pyridyl groups. Along the [011] plane, such mononuclear units related by 2-fold screw operation are interlinked by intermolecular O1—H1B···N6 interactions (Table 1) involving water ligands and pyridyl rings of 2-bpt to generate a 2-D net with simple (4,4) topology, (Batten *et al.*, 1998) as shown in Fig. 2. The dimension of the large grid of the net is 9.8005 * 9.8005 Å^2^. Furthermore, the hydrogen-bonded 2-D nets are interdigitated and interlayer π···π stacking interaction can be observed between the nearly parallel pyridyl of the 2-bpt molecules as expected, which can extend the structure to a 3-D supramolecular architecture (Fig. 3). The center-to-center and center-to-plane separations of the pyridyl groups are 3.809 and 3.499/3.294 Å (with a dihedral angle of 6.9°), respectively. What's more, the lattice ClO_4_^-^ anions are located in the interlayer space [a volume of 308.9 Å, 20.2% of the unit-cell volume as evaluated by *PLATON* (Spek, 2009)] and also hydrogen bonded to the 3-D aggregate *via* multiple O_water_—H···OClO_4_^-^ and N_amino_—H···OClO_4_^-^ interactions (Table 1).

### Experimental

To a methanol (10 ml) solution of 2-bpt (12.0 mg, 0.05 mmol) was added a water (5 ml) solution of Ni(ClO_4_)_2_.6H_2_O (18.0 mg, 0.05 mmol) with stirring, then a methanol (10 ml) solution of 5-Nipa (11.0 mg, 0.05 mmol) was added to the above mixture. After vigorous stirring for *ca* 20 min, the resultant solution was filtered and left to stand at room temperature. Pale-green block crystals suitable for X-ray analysis were produced by slow evaporation of the solvent for two weeks in a 52% yield (10.0 mg based on 2-bpt). Anal. Calcd for C_24_H_24_Cl_2_N_12_NiO_10_ (%): C, 37.43; H, 3.14; N, 21.83. Found (%): C, 37.40; H, 3.19; N, 21.91. IR (KBr, cm^-1^)): 3396*b*, 1708*w*, 1626*s*, 1568*m*, 1539*m*, 1484*m*, 1459*m*, 1424*m*, 1385*m*, 1350*m*, 1276*w*, 1144*vs*, 1088*vs*, 838*w*, 786*w*, 730*m*, 629*s*.

### Refinement

All H atoms were placed in geometrically calculated positions with C—H = 0.93 Å, N—H = 0.90 Å, and O—H = 0.85 Å, and included in the final refinement in the riding model approximation, with displacement parameters derived from their parent atoms [*U*_iso_(H) = 1.2*U*_eq_(C) and 1.5*U*_eq_ (N and O_water_)].

### Crystal data

**Table:**

[Ni(C_12_H_10_N_6_)_2_(H_2_O)_2_](ClO_4_)_2_	*F*(000) = 788
*M**_r_* = 770.16	*D*_x_ = 1.672 Mg m^−^^3^
Monoclinic, *P*2_1_/*n*	Mo *K*α radiation, λ = 0.71073 Å
Hall symbol: -P 2yn	Cell parameters from 2386 reflections
*a* = 9.9219 (15) Å	θ = 2.4–24.5°
*b* = 14.359 (2) Å	µ = 0.89 mm^−^^1^
*c* = 10.9220 (18) Å	*T* = 296 K
β = 100.560 (3)°	Block, pale green
*V* = 1529.7 (4) Å^3^	0.20 × 0.18 × 0.16 mm
*Z* = 2	

### Data collection

**Table:**

Bruker SMART CCD area-detector diffractometer	2686 independent reflections
Radiation source: fine-focus sealed tube	2171 reflections with *I* > 2σ(*I*)
graphite	*R*_int_ = 0.028
phi and ω scans	θ_max_ = 25.0°, θ_min_ = 2.4°
Absorption correction: multi-scan (*SADABS*; Bruker, 2001)	*h* = −10→11
*T*_min_ = 0.840, *T*_max_ = 0.870	*k* = −17→16
7639 measured reflections	*l* = −11→13

### Refinement

**Table:**

Refinement on *F*^2^	Primary atom site location: structure-invariant direct methods
Least-squares matrix: full	Secondary atom site location: difference Fourier map
*R*[*F*^2^ > 2σ(*F*^2^)] = 0.051	Hydrogen site location: inferred from neighbouring sites
*wR*(*F*^2^) = 0.154	H-atom parameters constrained
*S* = 1.07	*w* = 1/[σ^2^(*F*_o_^2^) + (0.090*P*)^2^ + 1.5989*P*] where *P* = (*F*_o_^2^ + 2*F*_c_^2^)/3
2686 reflections	(Δ/σ)_max_ < 0.001
223 parameters	Δρ_max_ = 1.12 e Å^−^^3^
0 restraints	Δρ_min_ = −0.40 e Å^−^^3^

### Special details

**Table:**

Geometry. All e.s.d.'s (except the e.s.d. in the dihedral angle between two l.s. planes) are estimated using the full covariance matrix. The cell e.s.d.'s are taken into account individually in the estimation of e.s.d.'s in distances, angles and torsion angles; correlations between e.s.d.'s in cell parameters are only used when they are defined by crystal symmetry. An approximate (isotropic) treatment of cell e.s.d.'s is used for estimating e.s.d.'s involving l.s. planes.
Refinement. Refinement of *F*^2^ against ALL reflections. The weighted *R*-factor *wR* and goodness of fit *S* are based on *F*^2^, conventional *R*-factors *R* are based on *F*, with *F* set to zero for negative *F*^2^. The threshold expression of *F*^2^ > σ(*F*^2^) is used only for calculating *R*-factors(gt) *etc*. and is not relevant to the choice of reflections for refinement. *R*-factors based on *F*^2^ are statistically about twice as large as those based on *F*, and *R*- factors based on ALL data will be even larger.

### Fractional atomic coordinates and isotropic or equivalent isotropic displacement parameters (Å^2^)

**Table:**

	*x*	*y*	*z*	*U*_iso_*/*U*_eq_	
Ni1	0.5000	0.0000	0.5000	0.0297 (2)	
Cl1	0.37983 (11)	0.36054 (8)	0.32882 (10)	0.0472 (3)	
O1	0.5877 (3)	0.12860 (19)	0.5625 (3)	0.0416 (7)	
H1A	0.6502	0.1302	0.6271	0.062*	
H1B	0.5281	0.1717	0.5556	0.062*	
O2	0.4370 (5)	0.2802 (3)	0.3892 (4)	0.0890 (14)	
O3	0.4030 (7)	0.3637 (7)	0.2091 (6)	0.184 (4)	
O4	0.2372 (5)	0.3650 (5)	0.3186 (6)	0.130 (2)	
O5	0.4382 (8)	0.4351 (3)	0.3942 (8)	0.203 (5)	
N1	0.5588 (3)	0.0213 (2)	0.3259 (3)	0.0330 (7)	
N2	0.3327 (3)	0.0682 (2)	0.4056 (3)	0.0328 (7)	
N3	0.2087 (3)	0.0979 (2)	0.4288 (3)	0.0365 (7)	
N4	0.2168 (3)	0.1192 (2)	0.2312 (3)	0.0318 (7)	
N5	0.1787 (4)	0.1333 (3)	0.1001 (3)	0.0424 (8)	
H5A	0.1399	0.0796	0.0693	0.064*	
H5B	0.1170	0.1795	0.0974	0.064*	
N6	−0.0420 (3)	0.2165 (3)	0.2012 (3)	0.0440 (8)	
C1	0.6805 (4)	0.0015 (3)	0.2978 (4)	0.0396 (9)	
H1	0.7459	−0.0283	0.3566	0.047*	
C2	0.7126 (4)	0.0244 (3)	0.1820 (4)	0.0456 (10)	
H2	0.7975	0.0085	0.1635	0.055*	
C3	0.6174 (5)	0.0706 (3)	0.0954 (4)	0.0468 (11)	
H3	0.6380	0.0884	0.0190	0.056*	
C4	0.4893 (4)	0.0901 (3)	0.1251 (4)	0.0414 (10)	
H4	0.4221	0.1200	0.0681	0.050*	
C5	0.4640 (4)	0.0642 (2)	0.2404 (3)	0.0324 (8)	
C6	0.3373 (4)	0.0829 (2)	0.2869 (3)	0.0305 (8)	
C7	0.1398 (4)	0.1292 (3)	0.3220 (4)	0.0332 (8)	
C8	0.0045 (4)	0.1734 (3)	0.3089 (4)	0.0353 (9)	
C9	−0.1653 (5)	0.2580 (3)	0.1895 (5)	0.0524 (11)	
H9	−0.2008	0.2869	0.1142	0.063*	
C10	−0.2413 (5)	0.2603 (3)	0.2819 (5)	0.0542 (12)	
H10	−0.3253	0.2908	0.2703	0.065*	
C11	−0.1904 (5)	0.2166 (3)	0.3918 (5)	0.0563 (12)	
H11	−0.2397	0.2174	0.4565	0.068*	
C12	−0.0655 (4)	0.1712 (3)	0.4068 (4)	0.0472 (11)	
H12	−0.0301	0.1402	0.4804	0.057*	

### Atomic displacement parameters (Å^2^)

**Table:**

	*U*^11^	*U*^22^	*U*^33^	*U*^12^	*U*^13^	*U*^23^
Ni1	0.0277 (4)	0.0354 (4)	0.0254 (4)	0.0013 (3)	0.0039 (3)	0.0021 (3)
Cl1	0.0438 (6)	0.0497 (6)	0.0437 (6)	0.0050 (5)	−0.0036 (5)	0.0001 (5)
O1	0.0389 (16)	0.0406 (15)	0.0433 (17)	−0.0022 (12)	0.0021 (13)	0.0004 (12)
O2	0.108 (3)	0.058 (2)	0.087 (3)	0.013 (2)	−0.018 (3)	0.014 (2)
O3	0.150 (6)	0.339 (11)	0.077 (4)	0.078 (6)	0.055 (4)	0.074 (5)
O4	0.061 (3)	0.203 (6)	0.129 (5)	0.035 (3)	0.028 (3)	0.020 (4)
O5	0.230 (8)	0.055 (3)	0.247 (8)	0.012 (4)	−0.159 (7)	−0.024 (4)
N1	0.0329 (17)	0.0362 (16)	0.0301 (17)	0.0002 (13)	0.0064 (14)	−0.0015 (13)
N2	0.0325 (17)	0.0390 (17)	0.0269 (16)	0.0028 (13)	0.0051 (13)	0.0010 (13)
N3	0.0338 (18)	0.0441 (18)	0.0308 (17)	0.0025 (14)	0.0038 (14)	0.0006 (14)
N4	0.0336 (17)	0.0352 (16)	0.0256 (16)	0.0004 (13)	0.0029 (13)	0.0006 (13)
N5	0.044 (2)	0.055 (2)	0.0268 (17)	0.0075 (16)	0.0039 (15)	0.0078 (15)
N6	0.0336 (18)	0.056 (2)	0.042 (2)	0.0059 (16)	0.0061 (15)	0.0106 (17)
C1	0.033 (2)	0.036 (2)	0.048 (2)	0.0017 (16)	0.0034 (18)	−0.0033 (18)
C2	0.036 (2)	0.055 (3)	0.049 (3)	−0.0033 (19)	0.016 (2)	−0.010 (2)
C3	0.053 (3)	0.056 (3)	0.036 (2)	−0.006 (2)	0.020 (2)	−0.002 (2)
C4	0.044 (2)	0.050 (2)	0.031 (2)	0.0007 (19)	0.0095 (18)	0.0035 (18)
C5	0.036 (2)	0.0314 (19)	0.0306 (19)	−0.0021 (15)	0.0085 (16)	−0.0006 (15)
C6	0.033 (2)	0.0306 (18)	0.0283 (19)	−0.0012 (15)	0.0056 (16)	0.0000 (15)
C7	0.031 (2)	0.0362 (19)	0.031 (2)	−0.0004 (15)	0.0037 (16)	−0.0015 (16)
C8	0.0298 (19)	0.037 (2)	0.039 (2)	−0.0018 (16)	0.0063 (16)	−0.0005 (17)
C9	0.041 (2)	0.053 (3)	0.061 (3)	0.009 (2)	0.004 (2)	0.014 (2)
C10	0.038 (2)	0.049 (3)	0.077 (4)	0.009 (2)	0.015 (2)	0.002 (2)
C11	0.045 (3)	0.065 (3)	0.066 (3)	0.001 (2)	0.029 (2)	−0.006 (3)
C12	0.043 (2)	0.059 (3)	0.042 (2)	0.002 (2)	0.012 (2)	0.003 (2)

### Geometric parameters (Å, °)

**Table:**

Ni1—N2^i^	2.037 (3)	N5—H5B	0.9000
Ni1—N2	2.037 (3)	N6—C8	1.334 (5)
Ni1—O1	2.101 (3)	N6—C9	1.346 (5)
Ni1—O1^i^	2.101 (3)	C1—C2	1.399 (6)
Ni1—N1	2.111 (3)	C1—H1	0.9300
Ni1—N1^i^	2.111 (3)	C2—C3	1.378 (7)
Cl1—O5	1.357 (5)	C2—H2	0.9300
Cl1—O3	1.369 (6)	C3—C4	1.397 (6)
Cl1—O2	1.396 (4)	C3—H3	0.9300
Cl1—O4	1.401 (5)	C4—C5	1.379 (5)
O1—H1A	0.8499	C4—H4	0.9300
O1—H1B	0.8500	C5—C6	1.465 (5)
N1—C1	1.330 (5)	C7—C8	1.468 (5)
N1—C5	1.348 (5)	C8—C12	1.378 (6)
N2—C6	1.323 (5)	C9—C10	1.367 (7)
N2—N3	1.369 (4)	C9—H9	0.9300
N3—C7	1.318 (5)	C10—C11	1.367 (7)
N4—C6	1.343 (5)	C10—H10	0.9300
N4—C7	1.366 (5)	C11—C12	1.383 (6)
N4—N5	1.426 (4)	C11—H11	0.9300
N5—H5A	0.9000	C12—H12	0.9300
			
N2^i^—Ni1—N2	180.00 (16)	C8—N6—C9	116.8 (4)
N2^i^—Ni1—O1	90.42 (11)	N1—C1—C2	121.7 (4)
N2—Ni1—O1	89.58 (11)	N1—C1—H1	119.1
N2^i^—Ni1—O1^i^	89.58 (11)	C2—C1—H1	119.1
N2—Ni1—O1^i^	90.42 (11)	C3—C2—C1	119.5 (4)
O1—Ni1—O1^i^	180.00 (7)	C3—C2—H2	120.2
N2^i^—Ni1—N1	101.04 (12)	C1—C2—H2	120.2
N2—Ni1—N1	78.96 (12)	C2—C3—C4	118.4 (4)
O1—Ni1—N1	89.94 (11)	C2—C3—H3	120.8
O1^i^—Ni1—N1	90.06 (11)	C4—C3—H3	120.8
N2^i^—Ni1—N1^i^	78.96 (12)	C5—C4—C3	118.9 (4)
N2—Ni1—N1^i^	101.04 (12)	C5—C4—H4	120.5
O1—Ni1—N1^i^	90.06 (11)	C3—C4—H4	120.5
O1^i^—Ni1—N1^i^	89.94 (11)	N1—C5—C4	122.4 (4)
N1—Ni1—N1^i^	180.000 (1)	N1—C5—C6	112.2 (3)
O5—Cl1—O3	110.3 (6)	C4—C5—C6	125.3 (4)
O5—Cl1—O2	107.8 (3)	N2—C6—N4	108.5 (3)
O3—Cl1—O2	110.8 (4)	N2—C6—C5	119.8 (3)
O5—Cl1—O4	109.5 (5)	N4—C6—C5	131.6 (3)
O3—Cl1—O4	105.5 (4)	N3—C7—N4	109.8 (3)
O2—Cl1—O4	113.1 (4)	N3—C7—C8	123.4 (3)
Ni1—O1—H1A	119.3	N4—C7—C8	126.7 (3)
Ni1—O1—H1B	111.7	N6—C8—C12	123.5 (4)
H1A—O1—H1B	116.3	N6—C8—C7	116.6 (3)
C1—N1—C5	119.0 (4)	C12—C8—C7	119.8 (4)
C1—N1—Ni1	126.2 (3)	N6—C9—C10	123.7 (4)
C5—N1—Ni1	114.7 (2)	N6—C9—H9	118.2
C6—N2—N3	109.0 (3)	C10—C9—H9	118.2
C6—N2—Ni1	113.7 (2)	C9—C10—C11	118.2 (4)
N3—N2—Ni1	137.1 (2)	C9—C10—H10	120.9
C7—N3—N2	106.3 (3)	C11—C10—H10	120.9
C6—N4—C7	106.4 (3)	C10—C11—C12	119.9 (4)
C6—N4—N5	124.0 (3)	C10—C11—H11	120.0
C7—N4—N5	129.3 (3)	C12—C11—H11	120.0
N4—N5—H5A	105.7	C8—C12—C11	117.8 (4)
N4—N5—H5B	101.1	C8—C12—H12	121.1
H5A—N5—H5B	112.2	C11—C12—H12	121.1
			
N2^i^—Ni1—N1—C1	−5.8 (3)	Ni1—N2—C6—N4	173.7 (2)
N2—Ni1—N1—C1	174.2 (3)	N3—N2—C6—C5	174.9 (3)
O1—Ni1—N1—C1	84.6 (3)	Ni1—N2—C6—C5	−9.6 (4)
O1^i^—Ni1—N1—C1	−95.4 (3)	C7—N4—C6—N2	2.2 (4)
N2^i^—Ni1—N1—C5	178.4 (2)	N5—N4—C6—N2	−172.0 (3)
N2—Ni1—N1—C5	−1.6 (2)	C7—N4—C6—C5	−174.1 (4)
O1—Ni1—N1—C5	−91.2 (3)	N5—N4—C6—C5	11.8 (6)
O1^i^—Ni1—N1—C5	88.8 (3)	N1—C5—C6—N2	8.2 (5)
O1—Ni1—N2—C6	95.9 (3)	C4—C5—C6—N2	−169.2 (4)
O1^i^—Ni1—N2—C6	−84.1 (3)	N1—C5—C6—N4	−175.9 (4)
N1—Ni1—N2—C6	5.9 (3)	C4—C5—C6—N4	6.7 (7)
N1^i^—Ni1—N2—C6	−174.1 (3)	N2—N3—C7—N4	0.6 (4)
O1—Ni1—N2—N3	−90.3 (4)	N2—N3—C7—C8	−175.7 (3)
O1^i^—Ni1—N2—N3	89.7 (4)	C6—N4—C7—N3	−1.7 (4)
N1—Ni1—N2—N3	179.7 (4)	N5—N4—C7—N3	172.1 (3)
N1^i^—Ni1—N2—N3	−0.3 (4)	C6—N4—C7—C8	174.4 (4)
C6—N2—N3—C7	0.8 (4)	N5—N4—C7—C8	−11.8 (6)
Ni1—N2—N3—C7	−173.2 (3)	C9—N6—C8—C12	−1.1 (6)
C5—N1—C1—C2	0.2 (6)	C9—N6—C8—C7	−179.2 (4)
Ni1—N1—C1—C2	−175.4 (3)	N3—C7—C8—N6	168.3 (4)
N1—C1—C2—C3	1.6 (6)	N4—C7—C8—N6	−7.3 (6)
C1—C2—C3—C4	−2.3 (6)	N3—C7—C8—C12	−9.9 (6)
C2—C3—C4—C5	1.4 (6)	N4—C7—C8—C12	174.5 (4)
C1—N1—C5—C4	−1.2 (6)	C8—N6—C9—C10	1.9 (7)
Ni1—N1—C5—C4	174.9 (3)	N6—C9—C10—C11	−1.1 (7)
C1—N1—C5—C6	−178.6 (3)	C9—C10—C11—C12	−0.4 (7)
Ni1—N1—C5—C6	−2.6 (4)	N6—C8—C12—C11	−0.3 (7)
C3—C4—C5—N1	0.4 (6)	C7—C8—C12—C11	177.7 (4)
C3—C4—C5—C6	177.5 (4)	C10—C11—C12—C8	1.1 (7)
N3—N2—C6—N4	−1.9 (4)		

Symmetry codes: (i) −*x*+1, −*y*, −*z*+1.

### Hydrogen-bond geometry (Å, °)

**Table:**

*D*—H···*A*	*D*—H	H···*A*	*D*···*A*	*D*—H···*A*
O1—H1A···O3^ii^	0.85	2.51	3.245 (8)	146
O1—H1A···O4^ii^	0.85	2.11	2.918 (7)	158
O1—H1B···O2	0.85	2.44	3.085 (5)	134
O1—H1B···N6^ii^	0.85	2.45	3.100 (5)	134
N5—H5A···O5^iii^	0.90	2.28	3.078 (7)	148
N5—H5B···N6	0.90	2.17	2.886 (5)	136

Symmetry codes: (ii) *x*+1/2, −*y*+1/2, *z*+1/2; (iii) −*x*+1/2, *y*−1/2, −*z*+1/2.
